# Screening Colonoscopy Association With Gastrointestinal Toxicity and Quality of Life After Prostate Stereotactic Body Radiation Therapy

**DOI:** 10.1016/j.adro.2025.101747

**Published:** 2025-02-22

**Authors:** Jonathan W. Lischalk, Vianca F. Santos, Brianna Vizcaino, Astrid Sanchez, Christopher Mendez, Kathleen Maloney-Lutz, Sam Serouya, Seth R. Blacksburg, Todd Carpenter, Moses Tam, Scott Niglio, William Huang, Samir Taneja, Michael J. Zelefsky, Jonathan A. Haas

**Affiliations:** a Department of Radiation Oncology, Perlmutter Cancer Center at New York University Langone Health - Long Island, New York, New York; b Department of Radiation Medicine, Lombardi Cancer Center at Georgetown University Hospital, Washington, DC; c Department of Gastroenterology, NYU Langone Hospital - Brooklyn, Brooklyn, New York; d Accuray Incorporated, Sunnyvale, California; e Department of Medicine, Perlmutter Cancer Center at NYU Langone Medical Center, New York, New York; f Department of Urology, Perlmutter Cancer Center at New York University Grossman School of Medicine, New York, New York

## Abstract

**Purpose:**

Screening colonoscopies (CS) performed before prostate stereotactic body radiation therapy (SBRT) allow for identifying synchronous malignancies and comorbid gastrointestinal (GI) conditions. Performing these procedures prior to radiation precludes the necessity of post-SBRT pelvic instrumentation, which may lead to severe toxicity and fistulization. We review compliance of CSs, incidence of GI pathology, and the impact of pretreatment CS findings on subsequent physician-reported toxicity and patient-reported quality of life (QoL).

**Methods and Materials:**

We reviewed an institutional database of patients treated for prostate cancer with SBRT including toxicity and QoL outcomes. A detailed review of pretreatment CS findings was reviewed including identification of diverticulosis, location of polyp resection, and presence of hemorrhoids. Pretreatment CS findings were then correlated with outcomes following SBRT.

**Results:**

Identification of comorbid GI conditions was a common event, with the presence of diverticulosis in 49.5% (n = 100), hemorrhoids in 67% (n = 136), and polyps in 48% (n = 98). More than half of patients with polyps removed had at least 1 removed from the rectosigmoid. Pretreatment CS did not introduce a delay in SBRT start date. Grade 1 toxicity was significantly lower in patients who underwent CS closer to the initiation of SBRT. There was no increased risk of physician-graded toxicity in the presence of diverticulosis, hemorrhoids, or polyps. Patient-reported GI QoL pattern in our screening cohort mimicked that seen in the previously published nonscreened population. There was no overt QoL detriment observed in patients who had GI pathology identified before SBRT.

**Conclusions:**

GI pathology identified in our elderly patient population was commonly identified on pretreatment CS. Screening CS may optimize bowel health for patients heading into radiation therapy. Toxicity and QoL for patients with GI pathologies identified on pretreatment CS do not preclude the delivery of prostate SBRT. We advocate for pretreatment CS in patients eligible prior to SBRT.

## Introduction

The National Cancer Institute ranks colon cancer as the third most diagnosed malignancy in both men and women in the United States. In the modern era, colon cancer has displayed a predilection for a younger patient population and as such, screening guidelines have been adjusted in recent years.^1^^,^^2^ Often, a prostate cancer (PCa) diagnosis can serve as an opportunity to optimize standard oncologic screening of more aggressive malignancies including lung and colorectal cancer. Moreover, in some cohorts, >3% of men with newly diagnosed PCa have a synchronous colorectal cancer diagnosed prior to treatment of the prostate malignancy.^3^ Given the risk of competing mortality, if a colorectal malignancy is diagnosed, treatment should be initiated before management of the PCa in most cases. As a result, literature from MD Anderson recommends performing a colonoscopy (CS) within 3 years of brachytherapy for PCa to avoid unnecessary posttreatment procedures.^3^

Obtaining CS prior to radiation therapy often precludes the necessity of pelvic instrumentation during the radiation follow-up (FU) period, which can oftentimes lead to excess toxicity. In the setting of a patient who recently underwent CS prior to stereotactic body radiation therapy (SBRT), the likelihood of developing a metachronous colorectal cancer shortly after prostate radiation is quite low. Prior literature in the GI community has recommended against biopsy of the anterior rectum after brachytherapy, except in very rare situations. However, similar recommendations have not clearly been made following SBRT. Given that rectal bleeding is not an uncommon manifestation of radiation proctitis, procedural investigation of said bleeding in less experienced hands can lead to severe and permanent damage of sensitive irradiated tissue and potentially fistulization if biopsied.^4^, ^5^, ^6^ The evaluation of radiation proctitis via CS may not change the natural history of expected endoscopic changes seen in the rectum that typically resolve with conservative management.^7^^,^^8^ This can be particularly true when higher doses of radiation are used and has been historically warned against in the setting of brachytherapy.^3^ Analogously, as high-dose SBRT becomes more ubiquitous in the radiation oncology community for the management of PCa, extreme caution should be placed on postradiation pelvic instrumentation at least within 1 year following SBRT.

In addition, CS identify comorbid GI conditions including diverticulosis and hemorrhoids, the management of which can be optimized with the help of a gastroenterologist before SBRT. The identification of such pathologies prior to radiation offers insight into a patient's underlying bowel health and risk for non–radiation-related GI exacerbations following treatment.^9^ Understanding the management of common GI conditions before initiating radiation therapy may help the patient optimize GI health as they head into pelvic radiation therapy and its sequelae. Moreover, there are limited data exploring toxicity and QoL following SBRT in patients with underlying diverticular or hemorrhoidal disease.

In the present study, we explore the compliance of CS in patients undergoing evaluation for PCa management prior to SBRT. We review the detailed GI findings on these pretreatment CS including diverticulosis, hemorrhoids, and polyp removal, as well as the association of these CS findings on the development of subsequent SBRT-related toxicity and patient-reported GI quality of life (QoL).

## Methods and Materials

### Patient eligibility

The local institutional review board (study #00001269) approved this single-institution review of patients treated with SBRT for PCa. We performed a retrospective review of patients with histologically-confirmed PCa treated with robotic SBRT from February 2021 to April 2024. All patients were evaluated by a radiation oncologist and deemed appropriate for SBRT. All patients underwent pretreatment diagnostic tests including clinical examination, prostate-specific antigen, and prostate biopsy. All patients underwent fiducial marker placement in the prostate approximately 1 week prior to simulation. Fiducial markers were used for inter- and intrafractional image guidance.

All patient CS results were reviewed in detail in concert with their evaluation by gastroenterology of comorbid GI medical conditions. CS procedural and pathology notes were obtained for detailed review, which allowed for CS findings to provide useful insights into pre-existing GI conditions. If the patient was eligible for a CS at the time of consultation or within 2 years of radiation treatment completion, the patient was then referred for a consultation with gastroenterology. Eligibility for CS was based on prior CS recommendations as well as standard United States Preventative Services Taskforce recommendations.

### Simulation, planning, and treatment delivery

All patients underwent computed tomography-based radiation treatment planning simulation (GE Optima 580). Magnetic resonance imaging of the prostate was obtained in most cases at the time of simulation and fused with the primary simulation computed tomography scan at the level of the fiducials to assist in target volume delineation. All patients underwent robotic SBRT with clinical target volume (CTV) which included the entire prostate and proximal or entire seminal vesicles. A 5-mm isometric expansion of the CTV with a tighter 3-mm posterior margin was used to create the Planning Treatment Volume (PTV). Dose calculations and planning optimization were performed using Accuray MultiPlan software. Dosimetric constraints for the normal structures were used based on institutional standards. Treatments were delivered using a robotic radiosurgical platform with prostate motion accounted for in the x-, y-, and z-plane.

### Toxicity and QoL analysis

After treatment delivery, patients were followed at standard intervals at 1 month, followed by every 4 months for 2 years, every 6 months for 3 years, and yearly thereafter. Physician-graded toxicity was performed by the same physician using Common Terminology Criteria for Adverse Event version 5.0. Patients received pre- and posttreatment Expanded Prostate Cancer Index Composite (EPIC) questionnaires at defined intervals to calculate patient-reported Health-Related Quality of Life. For the purposes of this project, only the GI EPIC domain was analyzed. The Health-Related Quality of Life score changes were considered meaningful according to minimally important difference (MID), set at half a standard deviation, as in prior publications.^10^^,^^11^ Descriptive statistics were first calculated to summarize the characteristics of the participants and the distribution of key variables. To assess the relationship between the given variables, Χ^2^ tests were employed. The calculated Χ^2^ value was compared to the critical value from the Χ^2^ distribution table at a significance level of 0.05.

This study used a longitudinal design to assess changes in QoL over time. Data were collected at 4 FU time points: < 6 weeks for the 1-month FU, between 3 and 5 months for the 4-month FU, between 6 and 9 months for the 8-month FU, and between 9 and 12 months for the 12-month FU. Participants were assigned to these time points based on their duration from baseline assessment. QoL scores were categorized into Bowel summary, Bowel subscale—Function, and Bowel Subscale—Bother. To assess the differences in QoL scores, we performed a *t* test to compare the mean scores between the groups. Subsequently, to account for the repeated measurements from the same subjects over time, we conducted a repeated-measures analysis of variance.

## Results

### Patient and tumor characteristics

From February 23, 2021 to April 25, 2024, 234 patients who underwent definitive radiation therapy for PCa had a mean age of 70 ± 7.7 years (range, 50-93 years). The median prostate-specific antigen level was 7.41 ng/mL. The National Comprehensive Cancer Network risk distribution was as follows: low (6.4%, n = 15), intermediate (64.5%, n = 151), high (23.9%, n = 56), regional (4.7%, n = 11), and metastatic (0.5%, n = 1). Of note, the majority (72%) of patients in this institutional cohort underwent pre-SBRT rectal spacer placement. Detailed information regarding patient and tumor characteristics can be found in Table 1.

**Table 1:** Patient and tumor cancer characteristics

	**%**	**n**
**Median age (y)**	70	234
**Age (range, y)**	50-93	
<60	10%	23
(60-70)	40%	95
>70	50%	116
**Median PSA (ng/mL)**	7.41	
**PSA (ng/mL)**		
<10	66%	155
(10-20)	26%	61
>20	8%	18
**Grade group**		
1	12%	29
2	45%	106
3	21%	50
4	14%	32
5	7%	15
Unknown	1%	2
**NCCN risk group**		
Low	6%	15
Intermediate	65%	151
High	24%	56
Regional	5%	11
Metastatic	0%	1
**Median prostate CTV (cc)**	77.82	

*Abbreviations:* CTV = clinical target volume; NCCN = National Comprehensive Cancer Network; PSA = prostate-specific antigen.

### Radiation treatment characteristics

The median total radiation dose was 3625 cGy delivered in 5 fractions (69.2%, n = 162) with the remainder receiving pelvic nodal irradiation with an SBRT boost to the prostate. The median duration of treatment was 9 days, with the duration ranging from 7 days to 51 days. A total of 139 patients (59.4%) received hormone therapy. Among the patients who received androgen deprivation therapy, 43.2% (n = 60) were recommended a duration of 4 to 6 months, 30.2% (n = 42) were advised for 18 months, and 26.6% (n = 37) were suggested a treatment length of 2 to 3 years. A total of 112 patients (47.8%) received a PI-RADS-directed 5-fraction SBRT microboost. More information regarding treatment characteristics can be found in Table 2.

**Table 2:** Treatment characteristics

ADT	%	n
Yes	59%	139
No	41%	95
**Recommended duration**		
4-6 mo	43%	60
18 mo	30%	42
2-3 y	27%	37
**Radiation total dose**		
**5 fractions:**		
3000 cGy	0.4%	1
3500 cGy	4%	9
3550 cGy	0.4%	1
3625 cGy	69%	162
*3 fractions:		
2100 cGy	21%	49
1950 cGy	3%	7
1800 cGy	0.4%	1
**2 fractions:**		
2500 cGy	1.8%	4
**Rectal spacer**		
Yes	72%	169
No	26%	60
Unknown	2%	5
**Prostatic microboost**		
Yes	49%	114
No	51%	120

*Abbreviation:* ADT = androgen deprivation therapy.

⁎ 3-fraction boost was delivered sequentially with conventionally fractionated pelvic nodal irradiation.

### Pretreatment colonoscopy characteristics

The vast majority (86%, n = 200) of patients received pre-SBRT CSs for screening purposes. Despite mandating pretreatment CS before the start of SBRT, there was no delay in treatment start date and in fact, patients who underwent CS had nominally lower median time from initial consultation to SBRT start date than those that did not (3.91 vs 4.70 months, *P* = .059). The median time from the date of CS to the treatment start date was approximately 4 months (range, 0-99 months). Nearly half of the patients (n = 114) had CS within 6 months of their treatment start date indicating the plurality of patients were eligible for CS, and preparation for PCa radiation therapy prompted the completion of this screening test.

For those patients who underwent a pretreatment CS, the presence of diverticulosis was observed in 49.5% (n = 100), hemorrhoids were present in 67% (n = 136), and polyps in 48% (n = 98). For those patients who underwent pre-SBRT CS within 6 months of treatment (n = 114), the presence of diverticulosis was observed in 44.7% (n = 51), hemorrhoids were present in 65.8% (n = 75), and polyps in 56% (n = 64). This analysis highlights the relatively high prevalence of these conditions in the screened cohort. Of those patients who had polyps removed, over half (57%, n = 55) had polyps removed in the rectosigmoid a median of 4.0 months prior to SBRT- where one would expect radiation therapy dose to be delivered during PCa treatment. More information regarding CS characteristics can be found in Table 3.

**Table 3:** Colonoscopy characteristics

Colonoscopy	%	n
Yes	86%	202
No	14%	32
**Median time to colonoscopy to prostate SBRT**		
<3 mo	32%	65
3-5 mo	23%	47
6-12 mo	11%	21
>12 mo	34%	69
**Diverticulosis**		
Yes	49%	100
No	51%	102
**Hemorrhoids**		
Yes	67%	136
No	33%	66
**Polyps**		
Yes	49%,	98
No	51%	104
**Polyp location**		
Rectum	26%	29
Sigmoid	37%	36
Descending colon	32%	31
Transverse colon	37%	36
Ascending colon	41%	40
Unknown	0%	2

*Abbreviation:* SBRT = stereotactic body radiation therapy.

### Colonic pathology and post-SBRT GI toxicity

**Figure 1:**
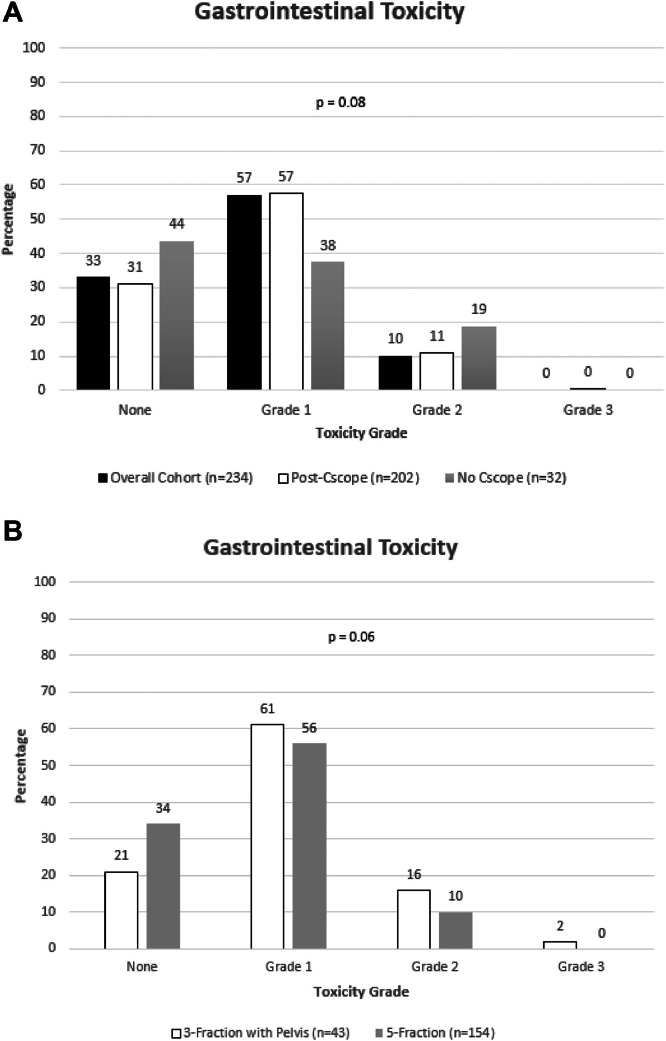
(A) Gastrointestinal toxicity among the entire cohort. No significant difference between cohorts. (B) Gastrointestinal toxicity in 5-fraction and 3-fraction with pelvis cohorts. Expected increased risk of toxicity in 3-fraction versus 5-fraction, although not significant (*P* = .06).

With a median FU of 1.25 years after SBRT, in those who underwent CS, the most common post-SBRT grade 1 GI toxicity included the following: diarrhea (45%), constipation (19%), and proctitis (14%). Gastrointestinal toxicity severity breakdown was as follows for the cohort: grade 1 (61%), grade 2 (10%), and grade 3 (1.4%). Overall, we did not see any significant differences in GI toxicity and positive colonic pathological outcomes between those who underwent a CS and those who did not prior to SBRT treatment (*P* = .08) (Fig. 1A). Interestingly, grade 1 GI adverse events were more common in patients who had an increased interval between CS and SBRT start date (497 vs 385 days, *P* = .035). However, this statistical difference disappeared when patients with grade 2 GI adverse events were analyzed (*P* = .78), although there were limited analyzable events in this cohort. Further breakdown of toxicity is displayed in Fig. 1.

**Figure 2:**
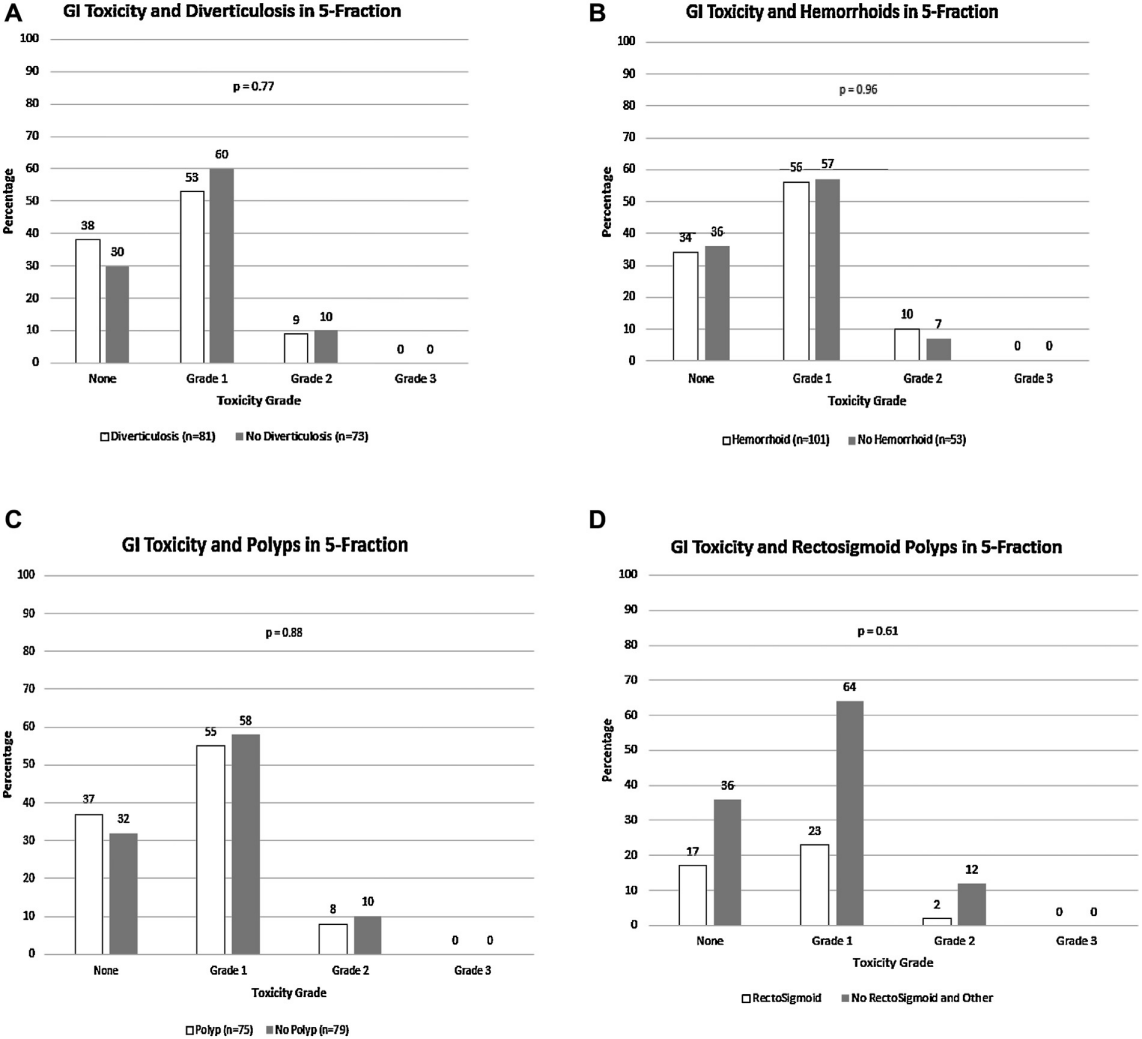
Gastrointestinal toxicity among those found to have on colonoscopy (A) diverticulosis, (B) hemorrhoids, (C) polyps, and (D) polyps localized to the rectosigmoid in 5-fraction cohorts. Presence of diverticulosis, hemorrhoids, any polyps, and polyps in the sigmorectum on CS did not result in an increased risk of SBRT-related toxicity. *Abbreviation:* CS = colonoscopy; SBRT = stereotactic body radiation therapy.

We then analyzed the association of documented CS identified colonic pathology and early Common Terminology Criteria for Adverse Event GI toxicity following SBRT. Although not significant (*P* = .06), there was an expected increased risk of toxicity in the 3-fraction with pelvis cohort relative to the 5-fraction prostate alone cohort (Fig. 1B). We specifically analyzed the 5-fraction cohort to determine if any pathologic findings yield an increased risk of toxicity. However, presence of diverticulosis, hemorrhoids, any polyps, and polyps in the sigmorectum on CS did not result in an increased risk of SBRT-related toxicity (Fig. 2A-D). Although limited by patient numbers in the 3-fraction with pelvis cohort, we similarly found no significant association between pathology and toxicity (Fig. E1A-D).

Finally, within the CS-screened cohort, for those patients who had evidence of hemorrhoids prior to treatment (n = 95), a flare in hemorrhoids occurred following SBRT in 11% of these patients with median time to flare of 1.22 months. The rate of hemorrhoidal development in those patients without evidence of hemorrhoids on a CS prior to treatment (n = 45) was much lower at 6% and occurred at a much longer interval from treatment, with a median time to occurrence of 9.7 months.

### Colonic pathology and post-SBRT GI EPIC QoL

**Figure 3:**
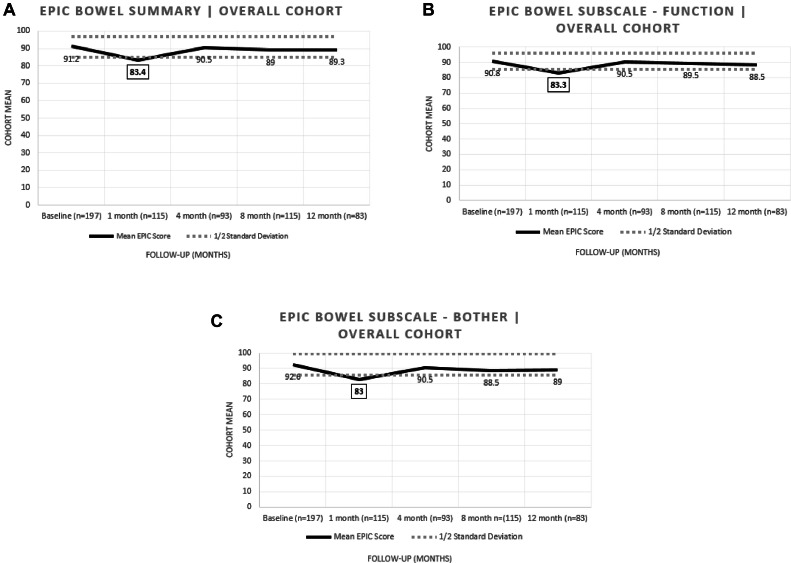
Overall cohort EPIC Quality of Life (A) Bowel Summary scores, (B) Bowel Subscale—Function scores, and (C) Bowel Subscale—Bother score. Epic Bowel Summary, Function, and Bother displayed deviations that were notable at 1-month follow-up (*P* < .001) with subsequent recovery by 4 months in each of the analyzed domains. *Abbreviation:* EPIC = Expanded Prostate Cancer Index Composite.

**Figure 4:**
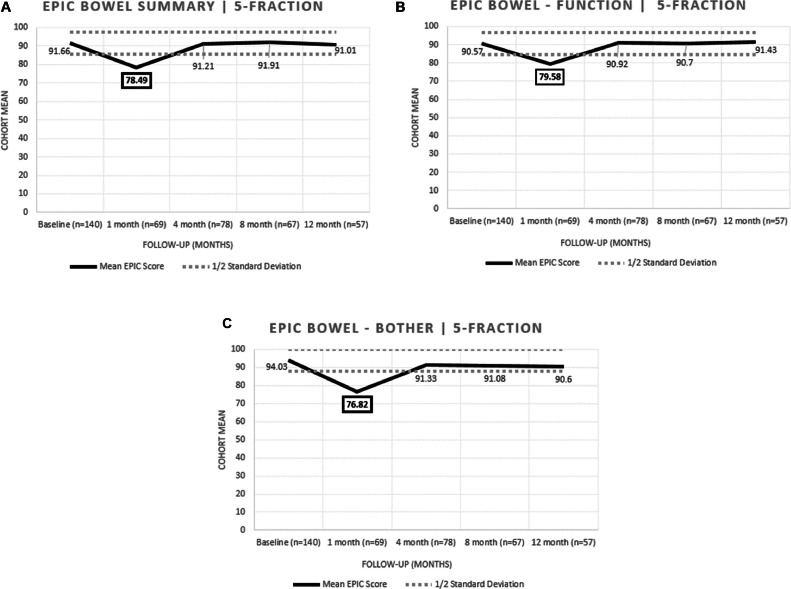
EPIC Quality of Life (A) Bowel Summary scores, (B) Bowel Subscale—Function scores, and (C) Bowel Subscale—Bother scores for those who completed a colonoscopy within the 5-fraction cohort. EPIC Bowel Summary, Function, and Bother displayed deviations that were notable at 1-month follow-up (*P* < .001) with subsequent recovery by 4 months in each of the analyzed domains. *Abbreviation:* EPIC = Expanded Prostate Cancer Index Composite.

Patient-reported GI QoL outcomes were utilized using standard EPIC questionnaires at baseline and following radiation treatment at specific time intervals detailed in the methods section. EPIC Bowel scores all displayed characteristics consistent with that reported in the literature (Figure 3, Figure 4).^10^, ^11^, ^12^ That is, EPIC Bowel summary, Function, and Bother displayed deviations that were notable at 1-month FU (*P* < .001) with subsequent recovery by 4 months in each of the analyzed domains. This bowel recovery at 4 months was sustained with longer FU out to 1 year. Of note, no MID was observed in patients without CS performed, although the cohort was severely limited by patient number (n = 21).

**Figure 5:**
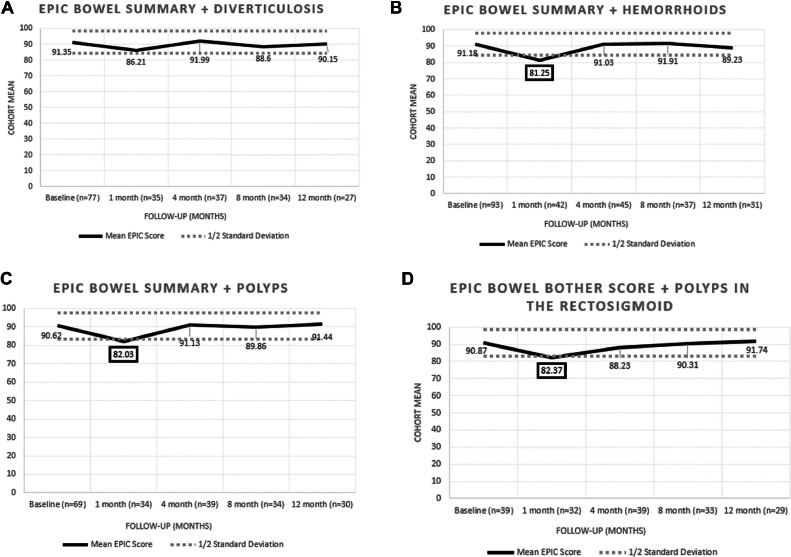
EPIC Quality of Life Bowel Summary Scores for those with (A) diverticulosis, (B) hemorrhoids, (C) polyps, and (D) polyps in the rectosigmoid within the 5-fraction cohort. Each cohort displayed a significant decline in bowel summary score at 1 month following SBRT with subsequent sustained recovery with longer follow-up. The only exception was those patients with diverticulosis who displayed a decline at 1 month post-SBRT that was not significant. *Abbreviations:* EPIC = Expanded Prostate Cancer Index Composite; SBRT = stereotactic body radiation therapy.

We then analyzed whether the presence of GI pathology in the 5-fraction cohort alone correlated with any abnormal deviations in post-SBRT QoL. There were no untoward deviations in EPIC GI QoL in patients who had presence of diverticulosis (n = 81), hemorrhoids (n = 98), any polyps (n = 75), and polyps in the rectosigmoid (n = 44). Each cohort displayed a significant decline in bowel summary score at 1 month following SBRT with subsequent sustained recovery with longer FU (Fig. 5). The only exception was those patients with diverticulosis who displayed a decline at 1 month post-SBRT that was not significant. Overall, QoL in the GI domain for those patients with GI pathology did not display more sustained deviations in MID beyond what would be expected in the absence of pathology.

## Discussion

In this single institutional review of patients undergoing pretreatment CS prior to prostate SBRT, compliance was overall excellent with 86% of patients undergoing CS. Identification of polyps was a common event and occurred in about half of our patient population, although no overt colorectal cancers were identified. Importantly, more than half of the patients who underwent polyp removal prior to SBRT had removal in the rectosigmoid, a sensitive location following radiation therapy. Moreover, identification of comorbid GI conditions including diverticulosis and hemorrhoids was observed in approximately 45% and 66%, respectively, of our screened cohort within 6 months of SBRT. As results of trials comparing conventionally fractionated radiation therapy with SBRT in the management of localized PCa have strongly supported the efficacy of SBRT, there has been a dramatically increased use of this modality in the treatment of PCa.^13^^,^^14^ Given the more ubiquitous use of this radiation modality, it is important to emphasize the significance of CS as has been historically done in the brachytherapy literature.

Overall, patients tolerated SBRT quite well with a very low rate of high-grade toxicity. Patients who underwent CS closer to the initiation of SBRT appeared to have a lower rate of grade 1 GI toxicity though this difference disappeared when analyzing grade 2 toxicity. One hypothesis for these results is that performing a CS closer to the initiation of SBRT offers the patient a detailed conversation with a gastroenterologist regarding bowel health and this may result in the reduction of low-grade GI toxicity following SBRT. Interestingly, there was no statistically significant association between identified colorectal diverticulosis, hemorrhoids, or polyps resection and the development of any graded toxicity. Moreover, rectosigmoid polyps resection prior to SBRT did not correlate with the development of an increased risk of SBRT-related toxicity. As such, identification of these common GI findings did not preclude SBRT delivery, at least with early FU.

In effort to characterize more subtle changes in GI QoL, patient-reported EPIC questionnaires were interrogated and demonstrated overall similar QoL patterns to previously reported outcomes in large cohorts in the absence of routine CS.^10^, ^11^, ^12^ That is, observation of a significant GI detriment 1 month after SBRT and subsequent sustained recovery with longer follow up. Performing a pretreatment CS did not result in a difference in GI QoL to that reported in the literature. Moreover, we identified no untoward sustained detriment in GI QoL when the presence of diverticulosis, hemorrhoids, or polyps was present on pretreatment CS.

Limitations of the present study include limited numbers of patients and relatively limited FU. Pathologic assessment of CS findings in this cohort also requires analysis to determine the spectrum of polyps identified in this patient population. It is valuable to note that hyperplastic polyps are commonly identified in the rectosigmoid and these lesions are often not resected at the time of CS. Similar to the historical recommendations in the brachytherapy literature, we recommend the completion of a CS prior to prostate SBRT in eligible patients for the following reasons: (1) identification of a comorbid colorectal cancer with a higher risk of mortality requiring upfront treatment; (2) preclusion of pelvic instrumentation during the sensitive FU period after SBRT during which manipulation of irradiated tissue may lead to severe long-term toxicity; and (3) based on the present data, a reduction in grade 1 GI toxicity following SBRT. Moreover, our data suggests that the removal of rectosigmoid polyps as well as identification of diverticular or hemorrhoidal disease does not preclude SBRT delivery, and with early FU no untoward GI toxicity or negative impact on patient-related QoL was observed in these populations. Future research should explore longer-term outcomes of gastrointestinal toxicity and QoL as it relates to underlying bowel pathology identified on screening CS.

## Conclusions

Pretreatment CS prior to prostate SBRT are prudent and allow assessment for synchronous malignancies and comorbid GI conditions. Completing a pretreatment colposcopy in eligible patients precludes the necessity of pelvic instrumentation during a sensitive FU period shortly after prostate SBRT. Polyp resection in the rectosigmoid as well as identification of diverticulosis and hemorrhoids are common events. The presence of such gastrointestinal pathology with early FU after prostate SBRT is not associated with increased risk of physician-graded toxicity or a detriment to patient-reported QoL.

## Disclosures

Jonathan Lischalk reports a relationship with Accuray that includes: speaking and lecture fees. Jonathan Haas reports a relationship with Accuray Inc that includes: consulting or advisory. Michael Zelefsky reports a relationship with Boston Scientific Corporation that includes: consulting or advisory. The other authors declare that they have no known competing financial interests or personal relationships that could have appeared to influence the work reported in this paper.

## Appendix Supplementary materials

Supplemental Materials Image, application 1
