# Supplementary material for: Screening Colonoscopy Association With Gastrointestinal Toxicity and Quality of Life After Prostate Stereotactic Body Radiation Therapy
Source: Adv Radiat Oncol. 2025 Feb 22;10(5):101747. doi: 10.1016/j.adro.2025.101747 (PMC12019482; doi:10.1016/j.adro.2025.101747)

**Supplemental Materials**

**Supplementary figure 1**: Gastrointestinal toxicity amongst those who A) with diverticulosis, B) hemorrhoid, C) polyps, and D) polyps localized to the rectosigmoid in 3- fraction with pelvis cohort.

A.


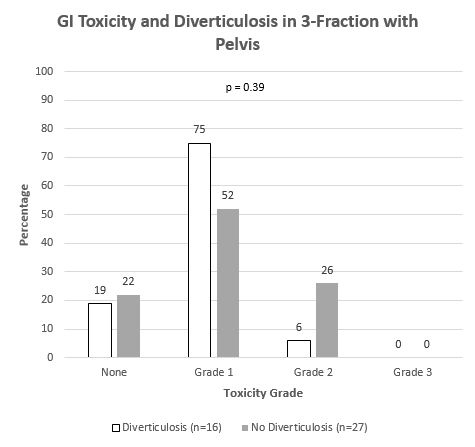


B.


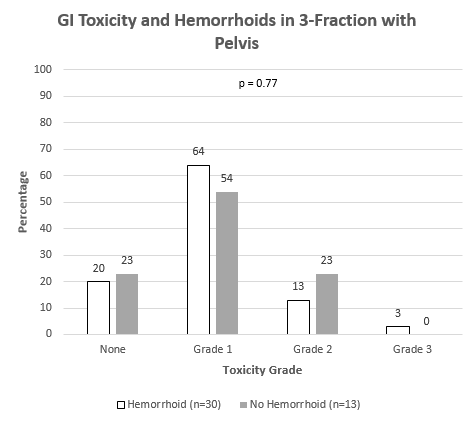


C.


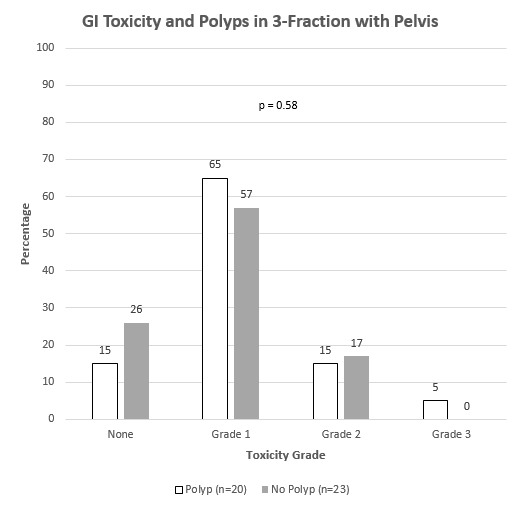


D.


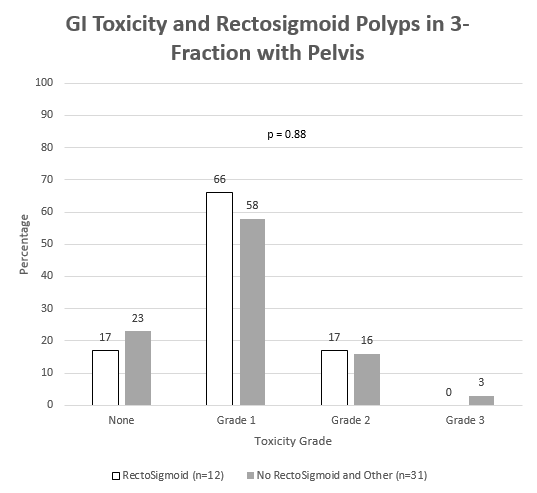

Supplement: Supplemental Materials [file mmc1.docx]
